# Experimental Comparison of the Reproductive Outcomes and Early Development of the Offspring of Rats Given Five Common Types of Drinking Water

**DOI:** 10.1371/journal.pone.0108955

**Published:** 2014-10-03

**Authors:** Hui Zeng, Wei-qun Shu, Ji-an Chen, Lin Liu, Da-hua Wang, Wen-juan Fu, Ling-qiao Wang, Jiao-hua Luo, Liang Zhang, Yao Tan, Zhi-qun Qiu, Yu-jing Huang

**Affiliations:** 1 Department of Environmental Hygiene, College of Preventive Medicine, Third Military Medical University, Chongqing, P. R. China; 2 The Lundberg-Kienlen Lung Biology and Toxicology Laboratory, Department of Physiological Sciences, Oklahoma State University, Stillwater, Oklahoma, United States of America; The University of Manchester, United Kingdom

## Abstract

Tap water (unfiltered), filtered tap water and processed bottled water (purified water, artificial mineralized water, or natural water) are now the five most widely consumed types of drinking water in China. However, the constituents (organic chemicals and inorganic ingredients) of the five waters differ, which may cause them to have different long-term health effects on those who drink them, especially sensitive children. In order to determine which type of water among the five waters is the most beneficial regarding reproductive outcomes and the developmental behaviors of offspring, two generations of Sprague–Dawley rats were given these five waters separately, and their reproductive outcomes and the developmental behaviors of their offspring were observed and compared. The results showed that the unfiltered tap water group had the lowest values for the maternal gestation index (MGI) and offspring's learning and memory abilities (OLMA); the lowest offspring survival rate was found in the purified water group; and the highest OLMA were found in the filtered tap water group. Thus, the best reproductive and offspring early developmental outcomes were found in the group that drank filtered tap water, which had the lowest levels of pollutants and the richest minerals. Therefore, thoroughly removing toxic contaminants and retaining the beneficial minerals in drinking water may be important for both pregnant women and children, and the best way to treat water may be with granular activated carbon and ion exchange by copper zinc alloy.

## Introduction

Global environmental and economic changes have led to the diversification of human drinking water. Traditional tap water is the most popular drinking water in the world. The addition of chlorine to tap water is one of the most common treatments to ensure its bacteriological quality. However, tap water remains susceptible to biological or chemical contamination [1]: if the water contains organic matter, this may produce disinfection by-products (DBPs), especially trichloromethane (THMs), in the water [2]–[4]. In addition, heavy metals such as lead and copper can be leached from pipes into the potable water stream [5]–[8]. Therefore, unpleasant tastes such as a chlorine flavor, DBPs and lead exposure in tap water may be the most common reasons driving people to choose alternative drinking water options such as bottled water or filtered tap water.

Bottled water's consumption has been steadily growing for the past 30 years. In 2011, the consumption was approximately 40,000 million liters in China (ranked number 1), 32,500 million liters in the United State of America (ranked number 2) and 262 billion liters in total around the world (90 countries) [9]. Three major types of bottled water are sold in Chinese groceries and supermarkets: bottled purified water, bottled mineralized water, and bottled natural water [10]. Bottled purified water, including distilled water, demineralized water, deionized water and reverse osmosis water, is usually tap water that has been treated by a series of filtration processes to remove nearly all minerals and electrolytes, disinfected by ozone or chlorine and finally packaged in a bottle (table 1) [11]. Thus, the purified water in theory is only H_2_O. However, the purified water tastes bad and may not quench thirst [12]. In order to improve the taste, small quantities of mineral salts such as potassium chloride and magnesium sulfate are added to the purified water, resulting in mineralized (or low-mineral drinking) water (table 1) [11]. Bottled natural water comes from high-quality underground or surface water sources. This water is also treated by serial filtration, usually disinfected by ozone and then packaged in bottles *in situ* (table 1) [11]. As such, bottled natural water generally contains certain amounts of minerals. Thus, it is clear that different bottled waters contain different minerals, and the mineral levels in these bottled waters are lower than those in the tap water.

**Table 1:** The drinking water treatment process for the five drinking waters in China.

	water treatment process
tap water	surface water → preliminary sedimentation → coagulation (aluminium polychlorid) → sedimentation → filtration → disinfected by chlorine → clean water tank → water pipes → user
bottled purified water	municipal tap water → quarts sand filtration → activated carbon → reverse osismis or nano filtration (0.0001 µm) → disinfected by ozone → packaged by plastic bottle(disinfect by chlorine) → user
bottled mineralized water	municipal tap water → quarts sand filtration → activated carbon → reverse osismis or nano filtration (0.0001 µm) → minerals added → disinfected by ozone → packaged by plastic bottle(disinfected by chlorine) → user
bottled natural water	surface/underground water → quarts sand filtration → activated carbon → ultrafiltration (0.001–0.1 µm)→ disinfect by ozone → packaged by plastic bottle(disinfected by chlorine) → user
filtered tap water	municipal tap water → activated carbon → KDF filtration → user

In a previous study that reported that drinking water is an important source of essential elements such as Ca and Mg [13], Sabatier suggested that magnesium and calcium in water is more bioavailable to a higher content (from 40% to 60%) than the magnesium and calcium obtained through diet because calcium and magnesium are mainly present as the simple ions Ca^2+^ and Mg^2+^ in water [14]. Furthermore, Gillies reported that tap water supplies 10% of the average individual's zinc intake [15]. Additionally, consumers want to have a drinking water option that has sufficient quantities of beneficial minerals but no pollutants, and filtered tap water may meet these requirements. Water filtration via a terminal water processor can not only remove chlorine and other impurities [16] but also significantly improve the taste and odor of public tap water. Therefore, it is suitable for home or anywhere where the water quality is poor. Currently, Chinese water pollution is widespread, and water filters are used in more and more residential buildings and private kitchens to improve public tap water quality. At present, more than 15% of the families in Beijing, Guangzhou and Shanghai have a household water purifier. Many materials can be used for water filtration: food-grade cocoanut active charcoal (CAC) and kinetic degradation fluxion (KDF) are the most popular in China. CAC can remove residual chlorine; KDF is a high-purity copper-zinc formulation that uses redox (oxidation/reduction) to remove chlorine, lead, mercury, iron, and hydrogen sulfide from water. The process also has mild anti-bacterial, algicidal, and fungicidal effects [17]–[18].

Therefore, the constituents in all five of these popular types of water are different and may have different biological effects in humans. Several studies have reported that many factors in drinking water have negative effects on human reproduction and development. For example, epidemiologic studies have reported that low calcium and magnesium intake from drinking water significantly increase the risk of delivering a very low birth weight baby [19]–[20]. Rats given water containing high levels of zinc have deficits in spatial and working memory [21]. Drinking purified water cannot obviously affect the rats' reproductive outcomes, but it can induce the occurrence of development retardation in the offspring [22]. Eliminating harmful effects on humans over their lifetime and in future generations is the ultimate goal of improving drinking water. However, up to now, there have been no comparative studies regarding the effects of drinking water on the reproductive system and offspring development. In this study, the reproductive and developmental outcomes of rats given the five types of water consumed most widely in China were compared to address which drinking water is the best for pregnant women and infants.

## Materials and Methods

### Ethics Statement

The Sprague–Dawley rats (150 females, 70∼90 g; 75 males, 110∼130 g) used in our experiments were obtained from the Laboratory Animal Center, Third Military Medical University (Chongqing, China) and were treated humanely according to the criteria outlined in the “Guide for the Care and Use of Laboratory Animals” prepared by the National Academy of Sciences. The protocol was approved by the Committee on the Ethics of Animal Experiments of Chongqing Experimental Animal Management Center. All serum sample collections were performed under sodium pentobarbital anesthesia, and all efforts were made to minimize suffering.

This study is part of a non-profit project supported by the National Natural Science Foundation of China. All necessary permits were obtained for the described field studies and approved by Chongqing Municipal Health Bureau. The study location is not privately owned, and the field studies did not involve endangered or protected species.

### Data Availability Statement

All data were uploaded as Data S1, accompanying the manuscript.

### Drinking water and diet

All the animals were acclimated to the laboratory environment for 1 week before the beginning of the study and had free access to food and water.

The tap water used was the municipal water of Chongqing city, which originated from the Yangzi River and was disinfected by liquid chlorine in the water treatment plant. The filtered tap water was municipal tap water filtered by a KDF-CAC-purifier. The tap water and filtered tap water were collected every day for the animals to drink. Three types of bottled water were purchased from supermarkets at a single time point, and each of the same type of bottled water was produced at the same time and was of a single origin. Furthermore, the quality guarantee period for the bottled water is 12 months, which ensured that the samples did not expire before the completion of the animal experiment. One box of bottled water was randomly selected from each type of bottled water to analyze the water quality parameters. The tap water and filtered tap water were collected once at the same time to analyze the water quality parameters. The water quality parameters were determined according to Chinese GB/5750-2006 [23].

The rat feed was prepared bi-monthly in the Laboratory Animal Center of the Third Military Medical University (license number: SCX 2007-018) and strictly followed the GB 14924-2001 in China for experimental animal feed nutrition (calcium 1.11%, magnesium 0.22%).

### Parent's reproductive procedure

**Figure 1:**
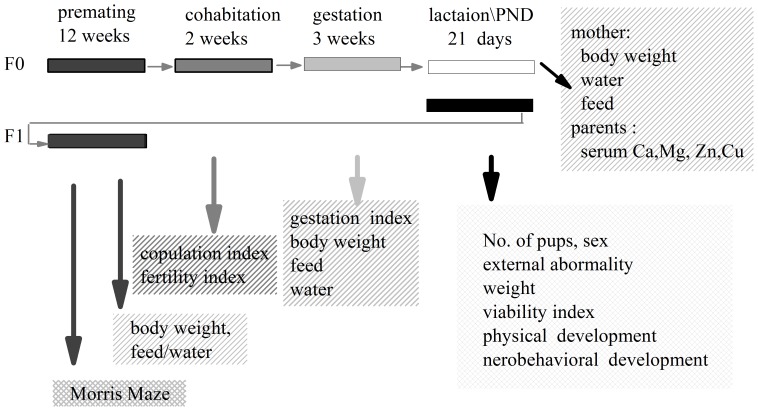
Schematic illustration of the study design (PND, postnatal day).

The animals were randomly divided into 5 groups, and each group was given one type of water from 28 days of age of the parents to 8 weeks of age of the pups (Fig. 1). The reproductive procedures were practiced according to the procedure modified from OECD 415 and GB15193.15-2003 in China [24]–[25]. At 119 days of age, two females were paired with one male (2∶1) from the same water group for 14 days until mating was confirmed by a copulatory plug or sperm in a vaginal rinse. The mating day was recorded as gestation day (GD) 0. The mated females were weighed on GD 0, 6, 12 and 18 and on lactation day (LD) 0, 4, 7, 14 and 21. Water and diet consumption were also recorded on the same days. The day of offspring birth was identified as postnatal day (PND) 0.

### Parents' serum calcium, magnesium, phosphorus, copper and zinc levels

Following lactation, parental rats were anesthetized by i.p. pentobarbital, and blood was collected by heart puncture into heparinized syringes. The total serum concentrations of calcium, magnesium phosphorus, and copper were determined in an Olympus AU 600 auto-analyzer(Japan) using the commercial kits of the same brand. Serum zinc was determined in an air–acetylene flame atomic absorption spectrometer (Puxi TAS-986, Peking).

### Parents' serum estradiol and testosterone levels

Serum estradiol and testosterone levels were determined in a radioimmunoassay counter (GC-911γ, Zhongjia photoelectric company) using commercial kits (Northern Beijing Institute of Biotechnology).

### Offspring studies

All the pregnant rats were allowed to give birth and nurture their offspring normally. On PND 0, the pups were examined for gross malformations, and the numbers of live and stillborn pups were recorded. Each litter was examined twice daily for survival. On PND 4, 8 surviving pups (four males and four females, if possible) were retained randomly, and the remaining pups were culled from each nest. Each pup was weighed on PND 0, 4, 7, 14 and 21.

All behavioral development parameters of the pups were assessed between 9:00 and 10:00 a.m. Pups were separated from the mothers for the time of observation and then immediately returned to their home cages. Pinna detachment, incisor eruption, eye opening, cliff avoidance and surface righting [26]–[27] were recorded according to the method previously described by our group [22].

The acquisition of spatial learning and memory was assessed via three components in the Morris water maze (MWM) (including hidden platform acquisition, probe trial and subsequent visible platform test) according to a modified version of the procedure of Morris [28]. Ten pups were chosen randomly from ten different litters per group for the MWM. On PND 28, all of them were tested in the MWM [22]. In the hidden platform acquisition test, the rat was given two trials a day for 5 days with an inter-trial period of 15 min, and the time to reach the escape platform was measured. Thus, the learning test refers to the time to reach a hidden platform on successive trials over 5 days. The probe trial was conducted on the sixth day. The platform was removed from the pool, and each rat was allowed to swim for 90 s in the water maze. The number of platform area crossings was recorded, and the memory test refers to the number of goal crossings (traversing the actual location of the escape platform). In the visible platform test, each rat was placed in the pool to find the visible platform in 90 s, and the time to find the platform and the swimming velocity were observed and measured.

### Statistical analysis

Following an assessment for homogeneity of variance, the data for quantitative and continuous variables (e.g., body weights) collected from all the rats were analyzed by one-way ANOVA. Data were transformed to achieve approximate normality if the data were not normally distributed. Data are presented as medians (interquartile range). For the hidden platform test, a repeated-measure-ANOVA was used to determine the significance of the difference among the groups. The frequency data were analyzed by a nonparametric χ^2^-test. The Pearson bivariate correlation analysis test was used to analyze the relationship between the levels of minerals (in water and serum) and reproductive or neurobehavioral parameters. Unless otherwise noted, the presented data are the mean values ± standard errors of the means (SEM). Statistical analyses were performed using SPSS 20.0 Statistical Software. In all the experiments, p<0.05 was considered to be statistically significant.

## Results

### The constituents of the five types of drinking waters

Table 2 shows that the levels of the constituents in the 5 waters were all within the levels established by the GB5749-2006 in China^29^ and the WHO guidelines (2011). The three bottled waters all contained relatively low levels of total dissolved solids (TDS) and total hardness (TH), indicating that the levels of inorganic components were lower in the bottled waters. Macro elements such as calcium, magnesium, and sodium were higher in the tap water and the filtered tap water than in the three bottled waters. Furthermore, the calcium and magnesium ratio was 1∶20 in mineralized water but 2∶1 in natural water and 4∶1 in tap water or filtered tap water. Zinc, another important component, was highest in tap water. Fluoride and nitrate concentrations were also higher in tap water and filtered tap water than in the three bottled waters. Arsenic is a toxic element in water, and its level in tap water reached the limit of GB5749-2006 in China. Chemical oxygen demand (COD), an indicator of the level of organic components, was the highest in tap water among five waters (Table 2). Trichloromethane, a conventional water quality index of the DBPs, was highest in bottled mineralized water and lowest in the filtered tap water. Perchloromethane, another conventional water quality index in Chinese GB5749-2006 that may originate from raw water, was found at high levels in three types of water.

**Table 2:** The water quality indices of the five drinking waters.

	bPW	bMW	bNW	FTW	TW	WHO Guideline (2011)	GB5749-2006 in China	LDL	Unit
pH	6.8	6.8	7.55	7.72	7.57	6.5–8.5	6.5–8.5	0	
TDS	1.2	10.9	87.2	291	229	<1000	<1000	0.1	mg/L
TH_CaCO3_	0.8	2.3	69.6	202.4	200.3	——	<450	0.05	mg/L
COD_Mn_	0.5	0.6	0.6	0.6	1.0	——	<3	0.05	mg/L
Potassium	<0.5	3.4	<0.5	2.1	2.5	——	——	0.5	mg/L
Sodium	0.1	0.1	0.1	17.0	12.4	——	200	0.1	mg/L
Calcium	0.04	0.02	0.04	40.4	52.9	——	——	0.01	mg/L
Magnesium	0.02	0.4	0.02	11.0	12.7	——	——	0.01	mg/L
Zinc	0.01	0.01	0.01	0.03	0.07	——	<1.0	0.01	mg/L
Copper	0.05	0.05	0.06	0.05	0.05	<1.0	<1.0	0.01	mg/L
Iron	<0.01	<0.01	<0.01	<0.01	0.13	<0.3	<0.3	0.01	mg/L
Mercury	<0.0001	0.0002	<0.0001	0.0001	0.0003	<0.006	<0.001	0.0001	mg/L
Arsenic	<0.01	<0.01	<0.01	<0.01	0.01	<0.01	<0.01	0.01	mg/L
Lead	<0.005	<0.005	<0.005	<0.005	<0.005	<0.01	<0.01	0.005	mg/L
Nitrite (nitrogen)	0.002	<0.001	0.001	0.026	<0.001	<0.9	<0.005	0.001	mg/L
Nitrate (nitrogen)	<0.5	<0.5	0.5	0.8	1.2	<11	<10	0.5	mg/L
Fluoride	<0.1	<0.1	<0.1	0.2	0.2	<1.5	<1.0	0.1	mg/L
Perchlormethane	0.0013	0.0009	0.0011	<0.0001	<0.0001	——	<0.002	0.0001	mg/L
Trichlormethane	0.021	0.033	0.015	<0.001	0.029	<0.3	<0.06	0.001	mg/L

Abbreviation: bMW, bottled mineralized water; bNW, bottled natural water; bPW, bottled purified water; COD, Chemical Oxygen Demand; FTW, filtered tap water; TDS, Total Dissolved Solids; TH, Total hardness; TW, tap water; WHO, world health organization; LDL, lowest detectable limit; —— not establishing guideline values.

### Parents' body weight, feed consumption and water consumption

No significant differences in body weight, water consumption or feed consumption were observed in maternal rats in the premating, mating, gestation and lactation periods (data are included in Data S1).

### Parents' reproductive parameters and serum levels of estradiol and testosterone

Table 3 shows that the maternal fertility index in the bPW group was significantly lower than that in the TW group, but the maternal gestation index in the TW group was statistically lower than in the other groups. There were no significant differences in the copulation index (male and female), fertility index (females) or the gestation length (females). Furthermore, there were also no significant differences in the levels of testosterone (male) and estradiol (females) among all groups (data are included in Data S1).

**Table 3:** The reproductive outcomes of the rat parents given the five drinking waters.

	bPW	bMW	bNW	FTW	TW	*P*
No. of rats (male/female)	30/15	30/15	30/15	30/15	30/15	
Copulation index (%)^a^, male	100 (15/15)	100 (15/15)	100 (15/15)	100 (15/15)	100 (15/15)	1.00
Copulation index (%)^a^, female	100 (30/30)	100 (30/30)	97 (29/30)	100 (30/30)	100 (30/30)	1.00
Maternal Fertility index (%)^b^	67 (20/30)*	100 (30/30)	83 (24/29)	73 (22/30)	100 (30/30)	0.00
Maternal Gestation index (%)^c^	100 (20/20)*	100 (30/30)*	100 24/24)*	100 (22/22)*	87 (26/30)	0.01
Gestation length (days)^d^	22 (0)	22 (0)	22 (0)	22 (0)	22 (1)	0.13

_a_Copulation index (%)  =  (no. of animals with successful copulation/no. of animals paired) × 100.

_b_Fertility index (%)  =  (no. of animals with pregnant/no. of animals with successful copulation) ×100.

_c_Gestation index (%)  =  (no. of females that delivered live pups/no. of pregnant females) ×100.

_d_Values are given as the median (interquartile range).

*p<0.05, statistically significant difference from TW group.

Abbreviation: bMW, bottled mineralized water; bNW, bottled natural water; bPW, bottled purified water; FTW, filtered tap water; TW, tap water.

### Parental serum concentrations of calcium, magnesium, phosphorus, copper and zinc

**Figure 2:**
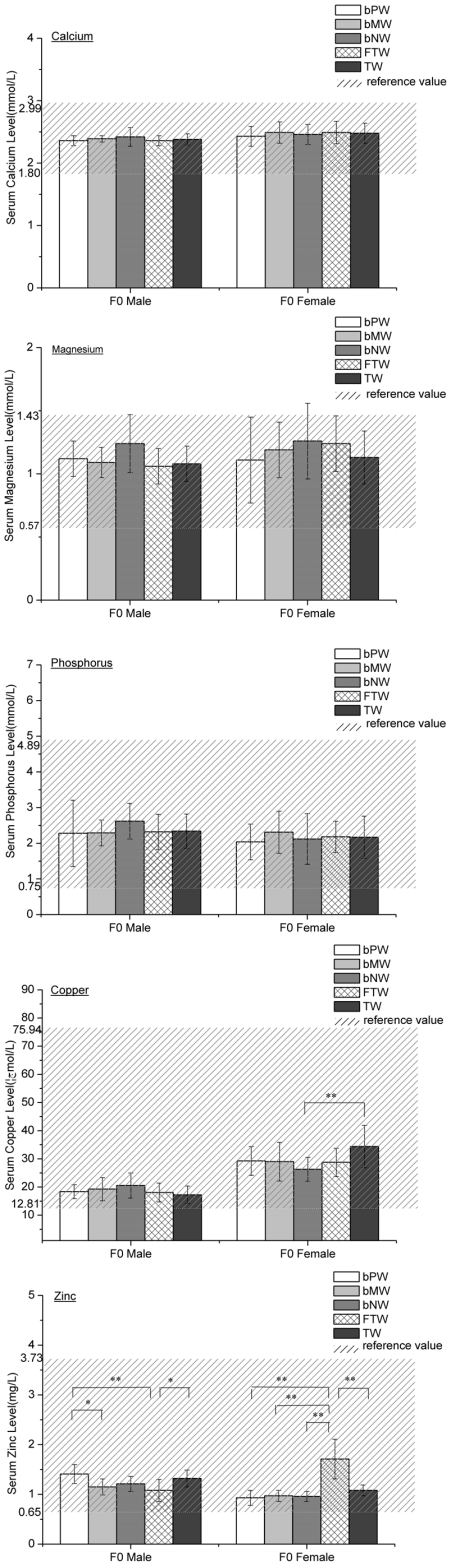
The serum mineral levels of the maternal rats after consuming the five waters (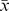
±s). Statistically significant differences between groups TW, bPW, bMW, bNW, and FTW are marked with asterisks: *p<0.05 and **p<0.01.

There were no significant differences in parental serum levels of calcium, magnesium or phosphorus among all five groups (Fig. 2). The maternal serum copper levels in the bNW group were statistically lower than in the TW group (Fig. 2). The paternal serum zinc levels in the FTW group were lower than in the TW and bPW groups, but the levels in the bPW group were statistically significantly higher than in the bMW group. Furthermore, the maternal serum zinc levels in the FTW group were the highest among the five groups (Fig. 2).

### Offspring's developmental parameters

**Figure 3:**
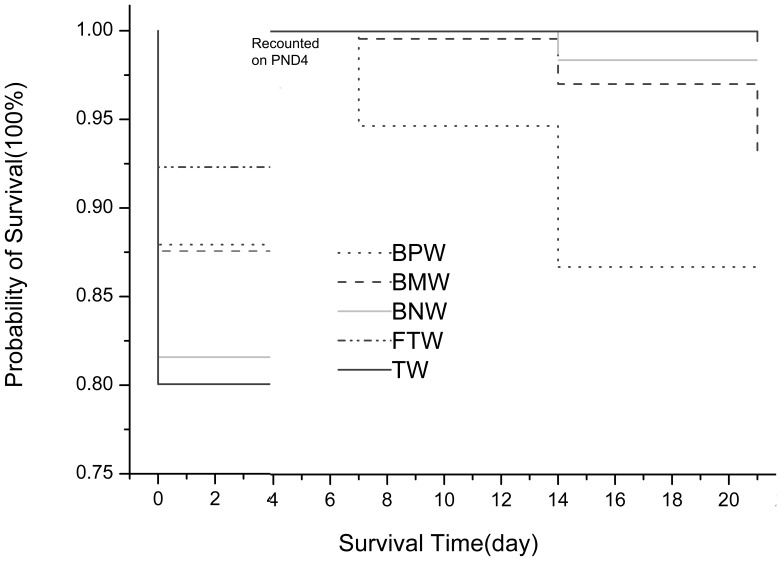
The survival plot of the F1 pups on lactation day 0, 4, 7, 14 and 21. Statistically significant differences between groups TW, bPW, bMW, bNW, and FTW on LD 0, LD 4 and LD 21 (p<0.05). The survival rats were recounted because 8 surviving pups (4 males and 4 females, if possible) were retained randomly, and the remaining pups were culled from each nest on PND 4.

On LD 0, there were no significant differences in external malformation, litter size, pup weight, sex ratio (data are included in Data S1) and survival rate (Fig. 3). On LD 4, survival rates in the TW and bNW groups were significantly lower than that in the FTW group (Fig. 3). On LD 21, the survival rate in the bPW group was the lowest among the 5 groups (Fig. 3). From LD 0 to LD 21, there were also no significant differences in pups' body weights, physiological development (pinna detachment, incisor eruption, and eye opening), and reflex development (surface righting and cliff avoidance reflex) (data are included in Data S1).

### Offspring's learning and memory ability after lactation

**Figure 4:**
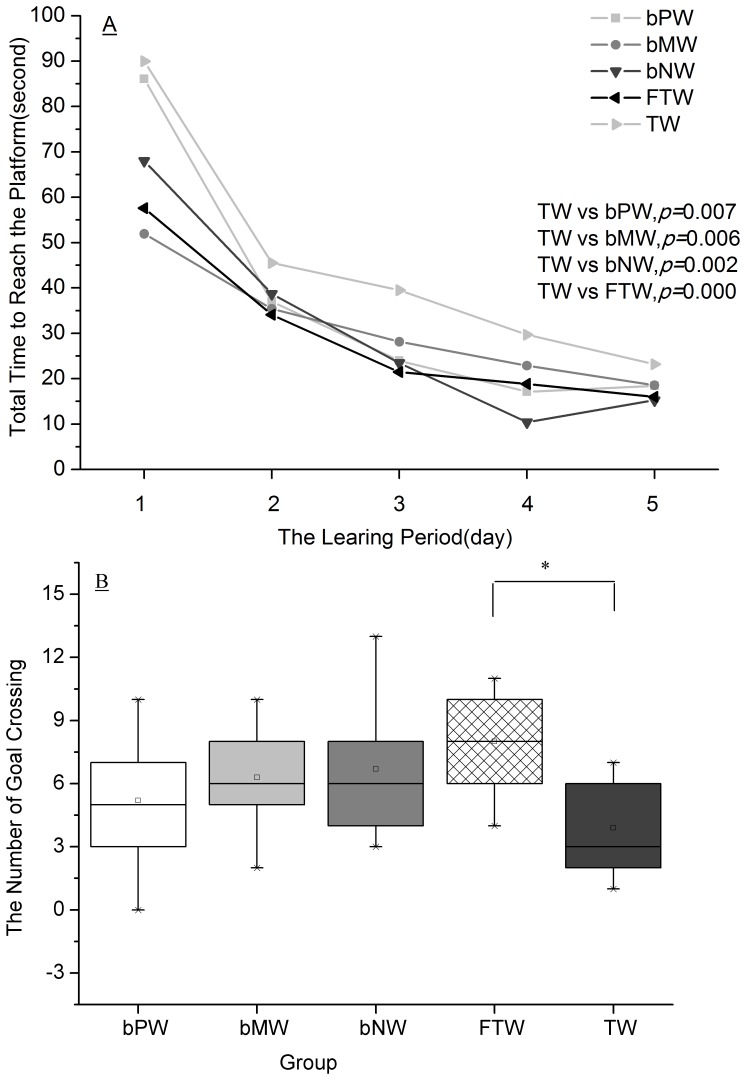
The pups' learning (A) and memory ability (B) in the place navigation test after the mothers drank each of the five waters (median). The data showed that the time to reach the platform of rat pups in the TW group was statistically longer than that of rat pups in the bPW, bMW, bNW and FTW groups, and the total number of rats to reach the platform in the FTW group was statistically higher than in the TW group. Statistically significant differences in the TW and FTW groups are marked with asterisks: *p<0.05.

During the learning period (days 1–5), the time to reach the platform in the place navigation test was shorter day by day in all groups (Fig. 4A), indicating that all the pups could generate space allocation memory regarding the platform. Based on the repeated measure ANOVA, the TW group showed a longer time to reach the platform than the bPW, bMW, bNW, and FTW groups (Fig. 4A). The pups' memory can be tested by the spatial probe test, and the number of goal crossings was recorded to denote the memory ability. Based on one-way ANOVA, the FTW group exhibited statistically significantly higher memory ability than the TW group (Fig. 4B). There were no significant differences in the visible platform trial (negative data not shown).

### The relationship of water constituents, serum mineral levels and reproductive and developmental parameters

Table 4 shows that the maternal gestation index was negatively associated with water COD (p = 0.005), zinc (p = 0.016), and arsenic levels (p = 0.000). It also showed that the pups' memory ability was also negatively associated with water COD (p = 0.012), arsenic (p = 0.009), and perchloromethane levels (p = 0.012) but was positively associated with maternal serum zinc levels (p = 0.027). Furthermore, maternal estradiol was negatively associated with perchloromethane (p = 0.042).

**Table 4:** Correlation analysis of the relationship among water constituents, serum mineral levels, maternal reproductive and pups' developmental parameters.

	Maternal Serum Calcium	Maternal Serum Magnesium	Maternal Serum Phosphorus	Maternal Serum Copper	Maternal Serum Zinc	Maternal Serum estradiol	Paternal serum Testosterone	Maternal Fertility Index	Maternal Gestation Index	Pup's Memory Ability
Water TDS	0.075	0.053	−0.002	0.163	0.675**	0.196	−0.047	0.020	−0.450	0.09
Water COD_Mn_	0.061	−0.070	0.020	0.356**	−0.009	−0.049	−0.172	0.669	−0.975**	−0.305**
Water Calcium	0.072	−0.035	0.003	0.308**	0.500**	0.141	−0.092	0.220	−0.738	−0.096
Water Magnesium	0.077	−0.025	0.007	0.291**	0.551**	0.157	−0.085	0.189	−0.683	−0.061
Water Zinc	0.055	−0.082	−0.001	0.372**	0.201	0.038	−0.133	0.437	−0.943**	−0.261
Water Copper	−0.042	0.148	−0.042	−0.260**	−0.243	−0.099	0.028	−0.059	0.250	0.118
Water Arsenic	0.034	−0.112	−0.004	0.381**	−0.079	−0.054	−0.150	0.567	−1.00**	−0.368**
Water Trichlormethane	−0.105	−0.011	−0.041	−0.256*	−0.597*	−0.157	0.104	0.715	−0.406	−0.010
Water Perchlormethane	0.009	−0.127	0.060	0.181	−0.652**	−0.237*	−0.130	−0.295	−0.001	−0.353*
Maternal Serum Calcium	1.00	0.718**	0.671**	0.229*	−0.047	0.038	—	0.144	0.049	−0.166
Maternal Serum Magnesium	0.718**	1.00	0.734**	0.088	−0.001	0.067	—	−0.101	0.442	−0.130
Maternal Serum Phosphorus	0.671**	0.734**	1.00	0.005	−0.086	0.075	—	−0001	0.378	−0.132
Maternal Serum Copper	0.229*	0.088	0.005	1.00	0.165	−0.154	—	0.879**	−0.826	−0.079
Maternal Serum Zinc	−0.047	−0.001	−0.086	0.165	1.00	−0.227	—	0.176	−0.225	0.316**

*, *p*<0.05,

**, *p*<0.01 indicate significant difference level.

No statistically correlation between other water constituents and pups' development parameters, between maternal serum elements and pups' development parameters (data not shown).

Maternal serum copper and zinc levels were significantly associated with water constituents, including calcium and magnesium. However, maternal serum calcium and magnesium levels had no correlation with water constituents. No correlation was observed between other water constituents and other maternal reproductive or pup developmental parameters.

## Discussion

### Water constituents

Different water treatment procedures may result in different water compositions. In this study, the data showed the different TDS levels in five types of water. There were no minerals or very low mineral levels in the bottled purified water and bottled mineralized water, which suggested that the “mineralized water” on the market appears not to actually be mineralized water. The calcium and magnesium ratio also indicated that the “mineralized” water did not meet the natural rule. The TDS and TH were also lower in bottled natural water, which indicated that the filtration treatment removed the majority of the minerals. In contrast, house water filters removed only minimal amounts of beneficial minerals but effectively removed toxic metals by ion exchange. It should be noted that the organic constituent was detected in all five waters. Trichloromethane and perchloromethane were the most common DBPs detected in all three bottled waters, and the levels were very similar, indicating that the main source of organic pollutants may be the plastic bottle and cap, which were always disinfected by chlorine. To our knowledge, COD is commonly used to indirectly measure the amount of organic compounds in water [29] because it is difficult or impossible to identify each contaminant in water, especially the trace organic contaminants. The present study showed that the COD of the tap water was the highest, although it did not exceed the GB19298-2003 and GB5749-2006 in China [30]–[31]. The high COD in tap water indicated that trichloromethane was not the only organic pollutant. Previous studies in our laboratory reported that 30 types of non-volatile organic pollutants were detected in the tap water that came from the same water plant, while 46 types of non-volatile organic pollutants were detected in the source waters, which were the Yangzi River, in 2000–2001 [32]; 50 types of non-volatile organic pollutants were detected in the same source water in the year 2005 [33]. The water treatment technology in that water plant has remained the same to the present day. However, because the pollution of the water source has not stopped, the number and variety of pollutants may increase. Fortunately, it is obvious that trichloromethane and other pollutants were effectively removed from the tap water through the use of a house water filter. Thus, the three bottled waters were just soft water with low TDS, the tap water was full of not only minerals but also pollutants, and the filtered tap water was full of minerals but with fewer pollutants. Furthermore, the bottled mineralized water did not meet the natural rule with regard to the ratio of calcium to magnesium.

### Water constituents exert effect on reproduction

Previous studies have shown that macro elements such as calcium and magnesium and trace elements such as zinc and copper have great impact on reproduction [34]–[36]. In the present study, the data showed varied levels of such mineral elements in the five drinking waters (Table 2) and the lowest female gestation index but a high fertility index in the TW group (Table 3). These results suggest that embryo implantation in early pregnancy may be affected. Macro elements in the body such as calcium, magnesium and phosphorus are fully supplied in the diet, but when the micro elements are marginally deficient in the diet, water becomes the main source [37]. In this study, the calcium level was 1.11% and the magnesium level was 0.22% in the rat diet, and those levels can fulfill the rats' requirements for growth and development. Therefore, the serum levels of calcium and magnesium were not significantly different between the 5 groups, and the correlation analysis also showed no significant relationship between the levels of water macro elements (calcium, magnesium and phosphorus) and reproductive or developmental parameters. Micro elements such as zinc may be derived from the galvanized plumbing materials. In the present study, zinc levels were highest in the tap water. Unfortunately, we did not determine the zinc content in the feed, as nutritional requirements may be fully met by the rats' feed. However, the data also showed that the serum zinc level was statically different in all 5 groups, and there were no correlations between the serum zinc level and the water zinc level. The correlation analysis also showed that the zinc level in water was significantly and negatively correlated to the maternal gestation index, although maternal serum zinc levels had no significant relation to the maternal gestation index. Serum zinc values vary diurnally, decrease after meals, and appear to be related to gender and age [37]. More than 90% of zinc is stored in the muscle, and only 10% is present in the serum. Because the zinc content of the muscle was not determined in the current study, we cannot rule out the possibility that the differences in the serum zinc levels are partly attributed to zinc mobilization from muscle tissue. It appears that there was a complicated link between zinc levels in the water and serum. The relationship between water zinc levels and reproductive index parameters requires further study. Thus, the micro elements such zinc, but the not macro elements in the water, may affect the rats' reproduction.

Clearly, toxic chemical elements such as arsenic exert negative reproductive effects [38]. In the present study, we found that only the arsenic levels in tap water reached the limit of the GB19298-2003 and GB5749-2006 in China. A correlation analysis showed that arsenic level in the water was negatively associated with maternal gestation index. However, the arsenic values were very close to each other, suggesting that arsenic levels may have not been sufficient to decrease maternal reproductive capacity in the TW group but may have increased the effects of other factors; these findings require further study.

Organic contaminants in water also have clear reproductive toxicity [39]–[40]. There was a negative correlation between water COD and maternal gestation index parameters. It may be possible that the lowest MGI in the TW group may have been the result of organic pollutants in the tap water other than trichloromethane, which was present in similar levels in each of the 4 waters. The organic pollutants including DBPs in the tap water may also exert an effect on reproductive function, as 40 types of trace organic pollutants have been detected in tap water, and the organic extract can increase endometrial thickness in rats [41]–[42]. How the mixture exerts these effects require further study.

### Water constituents exert effects on development

Previous studies have suggested that zinc can enhance learning and memory ability [43] and that organic pollutants [44] and arsenic [45]–[46] in drinking water can decrease learning and memory abilities. The results from the present study are consistent with these findings: organic pollutants and arsenic were the highest in the TW group, and the learning ability of the pups in the TW group was statistically lower than in the other groups; organic pollutants were the lowest and the maternal serum zinc level was the highest in the FTW, and the memory ability of the pups in the FTW group was the highest among the 5 groups. A correlation analysis also showed that the pups' memory ability was positively correlated to maternal serum zinc levels and negatively correlated to COD_mn_, arsenic and perchloromethane levels in water. Thus, the better memory ability in the FTW group may be due to the near absence of organic compounds or toxic metals and the rich mineral content in the filtered tap water. However, the reason for the lower learning ability in the TW group may be complicated because COD_mn_, arsenic and perchloromethane levels were all below the Chinese water standards GB5749-2006 and WHO Guidelines (2001) for tap water; in particular, the arsenic levels were very close in all five waters, which suggests that the effect on learning ability is most likely due to other pollutants we did not detect or a complex combination of all pollutants. These results require further study.

Survival rates on PND 4 and PND 21 can be used as indicators of the maternal nutritional status and maternal instinct. On PND 0, survival rates were not significantly different between the 5 groups. However, on PND 4, survival rates in all 5 groups decreased, and the survival rates in the TW and bNW groups were statistically lower than in the FTW group, which indicated that the maternal nutrition may have been deficient in the TW and bNW groups because the mothers nursed all pups. After PND 4, the pups were culled to 6–8 pups per litter in order to assure each mother had enough nutrition to nurse all pups. Therefore, the survival rate increased on PND 21. However, the survival rate in the bPW group was the lowest among the 5 groups, suggesting that maternal nutrition in the bPW group may have been insufficient.

Overall, among the five drinking waters, filtered tap water had the lowest levels of pollutants, had the highest hardness, was the richest in minerals, and showed the best benefit for maternal reproductive parameters and pups' development parameters. Removing toxic contaminants and maintaining minerals are both important for drinking water, especially when consumed by pregnant women and children. Granular activated carbon and ion exchange by copper zinc alloy may be the best way to treat water, but how water produced with these methods exerts beneficial effects needs further study. This result may have important implications for the selection of healthy drinking water and for water plants to optimize their treatment processes, as organic pollutants and toxic metals (arsenic) in drinking water may decrease maternal reproductive parameters.

## Supporting Information

Data S1 All data underlying the findings described in this manuscript.
(XLSX)
