# Correction: Experimental Comparison of the Reproductive Outcomes and Early Development of the Offspring of Rats Given Five Common Types of Drinking Water

**DOI:** 10.1371/journal.pone.0116244

**Published:** 2014-12-17

**Authors:** The PLOS ONE Staff

The authors are listed out of order. Please view the correct author order, affiliations, and citation here:

Hui Zeng^1^, Ji-an Chen^1^, Lin Liu^2^, Da-hua Wang^1^, Wen-juan Fu^1^, Ling-qiao Wang^1^, Jiao-hua Luo^1^, Liang Zhang^1^, Yao Tan^1^, Zhi-qun Qiu^1^, Yu-jing Huang^1^, Wei-qun Shu^1^*

^1^ Department of Environmental Hygiene, College of Preventive Medicine, Third Military Medical University, Chongqing 400038, P. R. China

^2^The Lundberg-Kienlen Lung Biology and Toxicology Laboratory, Department of Physiological Sciences, Oklahoma State University, Stillwater, Oklahoma, United States of America

Zeng H, Chen J-a, Liu L, Wang D-h, Fu W-j, et al. (2014) Experimental Comparison of the Reproductive Outcomes and Early Development of the Offspring of Rats Given Five Common Types of Drinking Water. PLoS ONE 9(10): e108955. doi:10.1371/journal.pone.0108955
